# The Efficacy of Anti-Arrhythmic Drugs in Children With Idiopathic Frequent Symptomatic or Asymptomatic Premature Ventricular Complexes With or Without Asymptomatic Ventricular Tachycardia: a Retrospective Multi-Center Study

**DOI:** 10.1007/s00246-021-02556-7

**Published:** 2021-01-30

**Authors:** Robin A. Bertels, Janneke A. E. Kammeraad, Anna M. Zeelenberg, Luc H. Filippini, Ingmar Knobbe, Irene M. Kuipers, Nico A. Blom

**Affiliations:** 1 Willem-Alexander Children’s Hospital–Leiden University Medical Center, Albinusdreef 2, P.O. Box 9600, Leiden, the Netherlands; 2 Sophia Children’s Hospital–Erasmus Medical Center, Dr. Molewaterplein 40, Rotterdam, the Netherlands; 3 Juliana Children’s Hospital–HAGA Hospital, Els Borst-Eilersplein 275, The Hague, the Netherlands; 4 VU Medical Center–Amsterdam UMC, De Boelelaan 1117, Amsterdam, the Netherlands; 5 Emma Children’s Hospital–Amsterdam UMC, Meibergdreef 9, Amsterdam, the Netherlands

**Keywords:** Premature ventricular complexes, Ventricular tachycardia, Children, Anti-arrhythmic drugs, Flecainide, Metoprolol

## Abstract

The aim of the study is to compare the efficacy of flecainide, beta-blockers, sotalol, and verapamil in children with frequent PVCs, with or without asymptomatic VT. Frequent premature ventricular complexes (PVCs) and asymptomatic ventricular tachycardia (VT) in children with structurally normal hearts require anti-arrhythmic drug (AAD) therapy depending on the severity of symptoms or ventricular dysfunction; however, data on efficacy in children are scarce. Both symptomatic and asymptomatic children (≥ 1 year and < 18 years of age) with a PVC burden of 5% or more, with or without asymptomatic runs of VT, who had consecutive Holter recordings, were included in this retrospective multi-center study. The groups of patients receiving AAD therapy were compared to an untreated control group. A medication episode was defined as a timeframe in which the highest dosage at a fixed level of a single drug was used in a patient. A total of 35 children and 46 medication episodes were included, with an overall change in PVC burden on Holter of -4.4 percentage points, compared to -4.2 in the control group of 14 patients. The mean reduction in PVC burden was only significant in patients receiving flecainide (− 13.8 percentage points; *N* = 10; *p* = 0.032), compared to the control group and other groups receiving beta-blockers (− 1.7 percentage points; *N* = 18), sotalol (+ 1.0 percentage points; *N* = 7), or verapamil (− 3.9 percentage points; *N* = 11). The efficacy of anti-arrhythmic drug therapy on frequent PVCs or asymptomatic VTs in children is very limited. Only flecainide appears to be effective in lowering the PVC burden.

## Introduction

Frequent premature ventricular contractions (PVCs) and asymptomatic ventricular tachycardia (VT) in children with structurally normal hearts are uncommon, less than 5% of school-aged children will have more than 50 PVCs per 24 h.[1–3] The exact prevalence is unknown, because symptoms are usually lacking. [4–6] Some children experience symptoms such as palpitations, but the PVC burden is usually not related to the severity of symptoms. In the pediatric population frequent idiopathic PVCs and asymptomatic VTs were regarded as benign conditions with a good prognosis. [1, 6, 7] However, recent data show that a high burden of PVCs (more than 20–30%) and asymptomatic VT can be associated with left ventricular dysfunction in children [8–10]. A causal relationship is suggested by the observations that left ventricular (LV) function usually recovers after effective treatment of ventricular arrhythmia [8, 9].

In general, frequent idiopathic PVCs and asymptomatic VTs in children do not require treatment. Guidelines recommend beta-blockers as first-line therapy of children with symptoms and of children in whom PVCs are thought to be causative of LV dysfunction [11, 12], with the exception of verapamil sensitive fascicular left ventricular VT. [13, 14]. Catheter ablation is a good alternative in older children after failure of AAD therapy or in case of side effects of medication. [11, 12] Literature data on efficacy and safety of AAD therapy in children are scarce and limited to small series. [4, 7, 8] In adults, recent guidelines recommend the Class IC drug flecainide as first-line therapy for idiopathic outflow tract VTs and symptomatic patients with PVCs [11, 15]. However, to date, there are no studies to support the use of flecainide as first-line therapy in children with frequent PVCs or asymptomatic VT. Therefore, the aim of this retrospective study is to compare the efficacy of anti-arrhythmic drugs for the treatment of frequent idiopathic PVCs and asymptomatic VT in children.

## Methods

In this retrospective multi-center study, patients were enrolled from databases of four university hospitals and one large regional referral hospital. Children above 1 year and under 18 years of age, suffering from symptomatic or asymptomatic frequent PVCs, with or without asymptomatic (non-) sustained VT were studied. An official waiver of ethical approval was granted from the local ethical committee.

Symptomatic or asymptomatic patients with a PVC burden of 5% [16] or more on 24-h Holter recording, with or without asymptomatic VT, and evaluated by consecutive Holter recordings were included. The decision to start AAD and the choice of AAD in the five different hospitals was based on institutional or physicians preference. Patients who received medication were included in four different groups based on the Vaughan Williams classification (Class I Na-channel blocker, Class II beta-blocker, Class III K-channel blocker, Class IV Ca-channel blocker). Patients without anti-arrhythmic medication were included as control group. Patients with structural heart disease, history of cardiac surgery, myocarditis, and inherited arrhythmia syndromes (based on family history, genetics, ECG criteria, echocardiographic and/or MRI findings) were excluded. Furthermore, patients with polymorphic PVCs or VTs, fascicular left ventricular tachycardia or prior radiofrequency ablation procedures with multiple lesions were excluded. Children receiving anti-arrhythmic drug therapy less than one year of age were excluded because of the increased likelihood of spontaneous resolution of PVCs or VTs in this age group.

Baseline characteristics including gender, age, weight, and symptoms (e.g., palpitations, dizziness, fatigue, chest pain or syncope) were recorded. ECGs and 24- or 48-h Holter recordings were evaluated to document PVC burden, presence of (non-)sustained VT, dominant QRS morphology (axis and bundle branch block pattern), and coupling interval (measured on ECG). Echocardiograms were reviewed to record the LV function and LV end-diastolic diameter (LVEDD), both were measured during sinus beats. LV dysfunction was defined as a shortening fraction of less than 28% [17, 18]. LVEDD was indexed to body-surface area (BSA) and Z-scores were calculated. During follow-up, radiofrequency ablation procedures were recorded.

A medication episode was defined as a timeframe in which the patient used only one type of anti-arrhythmic drug at a fixed dosage level. If the dosage was increased, only the medication episode with the highest dosage was included. Medication episodes with multiple medications at the same time were excluded. However, patients can be included in multiple groups if they received different AAD during separate periods of time. When the physician's records or correspondence indicated poor adherence to medication, the episode was excluded. Any medication that was prescribed only for pill-in-the-pocket strategy (in which the patients only takes AAD at the moment of complaints) was not included.

The efficacy of the medication in each episode was based on Holter recordings, documented before and after the start of medication. The exact change in percentage of PVCs on Holter recording was calculated and analyzed as continuous data. Statistical tests were performed using SPSS version 23.0 (IBM). The one-way ANOVA test was used to compare metric variables and the chi-square test for categorical variables. The changes in percentage of PVCs on Holter were tested using a gate-keeping procedure. At first an overall test was performed in a generalized linear mixed model, to determine if there was a difference between the five different groups. This was followed by a pairwise comparison between the different groups P-values of 0.05 or lower were considered significant.

## Results

Thirty-five Patients treated with AAD and 14 randomly selected patients without AAD (control group), evaluated by consecutive Holter recordings, were included. The 35 patients treated with AAD had 46 medication episodes, with a maximum of 3 episodes per child. In total, flecainide was prescribed 10 times, beta-blockers 18 times, sotalol 7 times, and verapamil 11 times. Children who received more than one type of anti-arrhythmic drugs during different medication episodes are represented in more than one group. The 14 randomly selected untreated patients had 20 episodes with consecutive Holter recordings. The baseline characteristics of the treated groups (before the start of medication) and control group were comparable regarding age at diagnosis, sex, weight, PVC burden, QRS morphology, coupling interval, and LV function (Table 1). The untreated group, as expected, had less symptoms, less VTs, and a lower LVEDD Z-score as compared to the treated groups. Symptoms in both the treated groups and the control group varied widely, consisting of palpitations, dizziness, fatigue, chest pain or syncope. The dosage of the medication prescribed was based on bodyweight and the way of administration depended on the patients age and available dosage form (i.e., multiple dosages per day or slow-release tablets). Therefore, the mean medication dose in mg per kg bodyweight per day is presented in Table 2.

**Table 1:** Baseline characteristics

	Controls (*N* = 20)	Flecainide (*N* = 10)	Beta-blocker (*N* = 18)	Sotalol (*N* = 7)	Verapamil (*N* = 11)	p-values
Male	9 (45%)	5 (50%)	9 (50%)	3 (43%)	7 (64%)	0.884
Age at diagnosis (years)	11 (4.7)	9 (5.6)	9 (5.0)	12 (2.7)	11 (3.0)	0.316
Weight (kg)	44 (23)	37 (24)	35 (20)	49 (11)	45 (15)	0.461
Symptoms	6 (30%)	4 (40%)	7 (39%)	6 (86%)	9 (82%)	0.016
PVC burden mean (SD),	20 (10)	29 (14)	24 (14)	33 (8)	24 (14)	0.190
median (min–max)	19 (7–40)	25 (11–56)	25 (5–57)	35 (20–45)	25 (5–52)	
(non-) sustained VT	2 (10%)	5 (50%)	8 (44%)	1 (17%)	6 (55%)	0.040
superior axis	3 (15%)	3 (30%)	2 (11%)	1 (14%)	0 (0%)	0.263
inferior axis	17 (85%)	6 (60%)	15 (83%)	6 (86%)	11 (100%)	
LBBB pattern	16 (75%)	7 (70%)	14 (78%)	6 (86%)	10 (91%)	0.799
RBBB pattern	4 (25%)	3 (30%)	4 (22%)	1 (14%)	1 (9%)	
Coupling interval (ms)	452 (56)	449 (113)	427 (98)	463 (71)	467 (71)	0.732
LV SF (%)	38 (5)	35 (6)	35 (4)	35 (8)	38 (4)	0.236
LV dysfunction	0 (0%)	2 (20%)	2 (11%)	1 (14%)	0 (0%)	0.238
LVEDD (mm)	44 (9)	46 (12)	42 (8)	49 (5)	45 (6)	0.458
LVEDD Z-score	0.79 (1.05)	2.13 (1.32)	1.20 (1.20)	1.72 (1.18)	0.92 (0.86)	0.032

Results are presented as N (%) and mean (SD). PVC burden = % of premature ventricular complexes per 24 h

*VT* = ventricular tachycardia, *LBBB* = left bundle branch block, *RBBB* = right bundel branch block, *LV* = left ventricle, *SF* = shortening fraction, *LVEDD* = left ventricular end-diastolic diameter

**Table 2:** Anti-arrhythmic drug dosage

Anti-arrhythmic drug	Mean dose
Flecainide (*N* = 10)	3.6 (0.8)
Metoprolol (*N* = 10)	1.9 (0.9)
Propranolol (*N* = 5)	1.7 (0.7)
Atenolol (*N* = 3)	1.2 (0.7)
Sotalol (*N* = 7)	3.0 (1.4)
Verapamil (*N* = 11)	4.2 (1.1)

Mean anti-arrhythmic drug dosage and standard deviation in mg/kg bodyweight/day

The mean interval between Holter recordings was significantly different between the five groups (p = 0,015), with the control group having the longest mean interval of 427 days (SD 310), the flecainide group 247 days (SD 253), the beta-blocker group 139 days (SD 175), the sotalol group 261 days (SD 435), and the verapamil group 145 days (SD 160).

The mean follow-up time in our study was 3.8 years, with no statistically significant differences between the five groups (p = 0.924): 4.2 years for the control group (SD 3.6), 3.4 years (SD 1.8) for children in the flecainide group, 3.4 years (SD 3.2) in the beta-blocker group, 4.0 years (SD 2.7) in the sotalol group, and 3.6 years (SD 1.6) in the verapamil group.

**Fig. 1:**
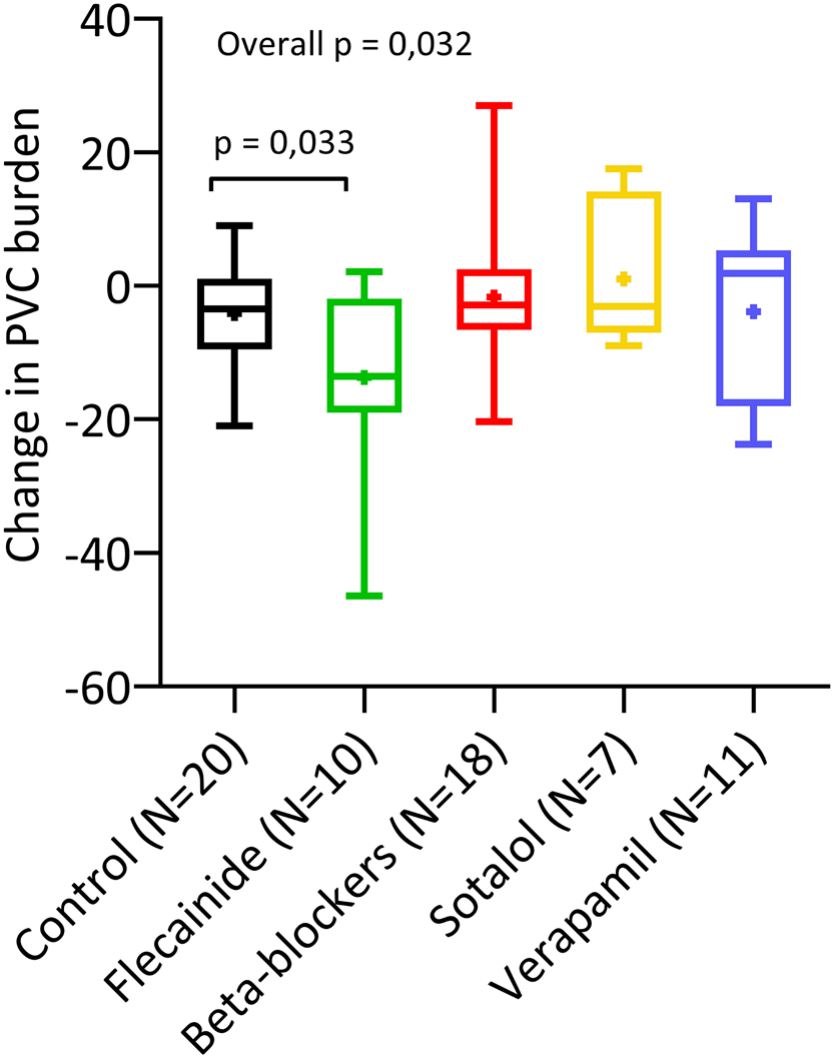
Mean change in PVC burden. Central Illustration: Mean change in PVC burden on Holter in percentage points: mean (+), median, upper and lower quartile, and minimum and maximum (Color figure online)

Overall, the mean reduction in PVC burden on Holter recordings was − 4.4 percentage points (range − 46 to + 27 percentage points) in the patients treated with AAD, compared to − 4.2 percentage points (range − 21 to + 9 percentage points) in the control group. The reduction in PVC burden of the 5 groups is presented in Fig. 1. There was no relation between age and PVC burden in a linear regression model (*p* = 0.527). Patients in the flecainide group had a mean reduction of -13.8 percentage points (*N* = 10), in the beta-blockers group -1.7 percentage points (*N* = 18), in the sotalol group + 1.0 percentage points (*N* = 7) and in the verapamil group -3.9 percentage points (*N* = 11). The overall test from the generalized linear mixed model indicated significant differences in reduction of PVC burden between the five groups (four treatment groups plus control group) (*p* = 0,032). Looking at the pairwise comparisons of the treated patients versus the control patients, only the difference between the flecainide group and the control group was significant (*p* = 0,033).The four treatment groups were also tested in a generalized linear mixed model without the control group. In this overall test there was a significant difference between the four treatment groups (*p* = 0,023).

The groups were too small to be able to perform further statistical analyses of subgroups with different dosages of medication. One patient with LV dysfunction had -46 percentage points PVC reduction on flecainide therapy and full recovery of LV function. The PVC reduction was confirmed on multiple Holters, returned after discontinuation of flecainide and resolved again after restarting flecainide. Catheter ablation was not performed because during electrophysiology study the PVC focus was located deep in the papillary muscle.

During follow-up, a catheter ablation procedure was performed in 14 patients with a mean age of 14 years (SD 3.4 years). Indications for ablation were symptoms in combination with failure of medication, and/or LV dysfunction. Of these procedures 11 were successful, with the focus of the PVCs in the right ventricular outflow tract (RVOT), at the right ventricular (RV) free wall or tricuspid annulus. Unsuccessful ablation sides were at the papillary muscle or LV summit.

## Discussion

To our knowledge, this is the first study to compare the efficacy of four types of anti-arrhythmic drugs in a group of children with frequent symptomatic or asymptomatic PVCs with or without asymptomatic VT to a control group. The main findings are: (1) the overall effect of anti-arrhythmic drugs (AAD) on frequent PVCs was very limited, with a mean change of the PVC burden on Holter recording of − 4.4 percentage points, compared to a control group with a change of − 4.2 percentage points; and (2) flecainide was the only AAD that significantly reduced the PVC burden (− 13.8 percentage points) compared to other classes of AAD and the control group. Since flecainide appears to be the only effective anti-arrhythmic drug for the treatment of frequent PVCs, we argue that there is reason to use flecainide as first-line AAD therapy if there is an indication to treat PVCs or non-sustained VT in children, which is contrary to current clinical practice.

In this study beta-blockers were the most frequently prescribed anti-arrhythmic drugs. This corresponds with a survey on AAD therapy preferences performed prior to this study, among 17 pediatric cardiologists from the participating centers. More than 70% of the respondents indicated that beta-blockers were their first-line medication, which is in accordance to international guidelines. [2, 12] However, evidence for these guidelines in children is scarce. Table 3 summarizes the results of a review of the literature, which we performed by a PubMed search for AAD therapy in pediatric patients with premature ventricular complexes or ventricular tachycardia. Thus far, only small retrospective series are published, none of the studies is large enough to reach statistical significance. As expected, beta-blockers are the most commonly used AAD, but treatment with all classes of AAD is described. Most series only report patients with VT, of which the study by Pfammatter et al. in 1999 was the largest. They reported a retrospective series of 73 children with VT, in which beta-blockers and sotalol were administered the most, with a response rate of 35% and 62%, respectively. Both class Ic drugs propafenone and flecainide had a response rate of 65%. Two other smaller studies also included patients with PVCs. A series on drug efficacy in 28 children with > 5% PVCs was published by Kakavand et al. (2010) and observed that only atenolol and flecainide appeared to be effective [8]. Heusch et al. (1994) found that propafenone (Class 1C) was effective in two out of five children with PVCs (4 patients) or VT (1 patient), and a structurally normal heart [19].

**Table 3:** Review of available evidence of efficacy of AAD in the pediatric age group

Year of publication First author	Age	Type of ventricular arrhythmia	AAD used	# patients using AAD	# patients with successful treatment (%)	Effect based on
2013 Collins [13]	Mean 10.0 yrs (± 5.1 yrs)	LVT	Ca-channel blockers	92 fascicular	73 (80)	Conversion to sinus rhythm or prevention of arrhythmia recurrence
			Ca-channel blockers	5 non-fascicular	2 (40)	
2010 Wang [4]	Mean 6.7 yrs (0 to 17.9 yrs)	VT	Procainamide	2	0 (0)	Improvement experienced by patients on the specified medication
			Mexiletine	4	3 (75)	
			Propafenone	8	4 (50)	
			Flecainide	1	1 (100)	
			Beta-blockers	15	11 (73)	
			Amiodarone	6	6 (100)	
			Sotalol	7	2 (29)	
			Ca-channel blockers	18	16 (89)	
2010 Levin [25]	Median 1 day (1–275 days)	VT	Procainamide	4 (2 incl Propranolol)		The time to ventricular tachycardia resolution
			Lidocaine	13		
			Mexiletine	6 (5 incl Propranolol)		
			Propranolol	11		
			Amiodarone	7		
			Overall	31	19 (61)	
2010 Kakavand [8]	Mean 13 (± 5 yrs)	PVC > 5%	Mexiletine	1	0 (0)	Not described Case presented of flecainide effect with decrease in PVC burden of 60% to < 1%, and recurrence of PVCs after outgrowing dosage
			Flecainide	1	1 (100)	
			Beta-blockers	13	Atenolol effective	
			Class IV	1	0 (0)	
			Digoxin	3	0 (0)	
2005 Iwamoto [7]	Mean 11 yrs (5 to 15 yrs)	VT	Mexiletine	6 (4 incl BB)		The decrease in VT episodes of more than 90% on Holter monitoring
			Beta-blockers	13		
			Verapamil	7 (3 incl BB)		
			Overall	26	20 (77)	
1999 Pfammmatter [6]	Mean 5.4 yrs (0.1 to 15.1 yrs)	VT	Propafenone	19	12 (65)	The lack of evidence of recurrences clinically and during Holter monitors
			Flecainide	6	4 (65)	
			Beta-blocker	23	8 (35)	
			Amiodarone	18	16 (89)	
			Sotalol	21	13 (62)	
			Verapamil	8	7 (88)	
			Overall	73	26 (35)	
1996 Davis [26]	Median 0.2 yrs (1 day to 4.8 yrs)	VT	Propafenone	29	2	Resolution of VT
			Beta-blocker		8	
			Amiodarone		6	
			Other		4	
1995 Tsuji [27]	Mean 9.3 yrs (1 mnth to 18 yrs)	VT	Beta-blockers	28	16 (57)	The complete absence of VT in at least 2 consecutive Holter, ECG or treadmill exercise tests
			Class IV	14	11 (79)	
			Propafenone	6	4 (67)	
1994 Heusch [19]	Mean 34 mnths (1 day to 16 yrs)	PVC (4) / VT (1)	Propafenone	5	2 (40)	The complete disappearance of VT and reduction of PVCs to monomorphic extra systoles

In the adult population with frequent PVCs there is more evidence for the efficacy of anti-arrhythmic drugs. In an open-label cross-over trial in 84 patients with idiopathic PVCs, Stec et al. (2012) showed that 42% responded to propafenone, 15% to verapamil, and 10% to metoprolol (*p* < 0.01). [20] Their results of low response rates match the results of this study; the high response to propafenone supports our findings that flecainide may be more effective than other classes of anti-arrhythmic drugs. [20] Gill et al. (1992) performed a cross-over study in 23 patients with right sided VT, who were serially prescribed verapamil, sotalol, and flecainide. [21] All three drugs caused a partial response documented on Holters, and authors concluded that sotalol was most effective, although no significant differences were found. [21] Krittayaphong et al. (2002) report in a placebo-controlled trial in 52 patients that atenolol significantly decreased symptoms and PVC count, from 24.082 to 16.153 per 24 h. [22] According to their criteria, the response rate was 24% in the atenolol group. Although placebo did not reduce the PVC count, it significantly decreased symptoms, demonstrating a strong placebo effect. However, a recent study by Hamon et al. (2019) describes that in a subgroup of patients the burden of PVCs can even be increased by beta-blockers, since usually PVCs are reduced at higher heart rates [23]. This might explain why the increase of PVCs in our study group is highest in the beta-blocker group. An extensive meta-analysis published in 1990, including 95 studies and 2989 patients, reported that class IC drugs and amiodarone were significantly more effective in treating ventricular ectopy [24]. However, 82% of the patients had a history of cardiovascular disease and, therefore, are difficult to compare to the population with idiopathic PVCs.

This retrospective study has a number of limitations. Although it is the largest series of children reported with symptomatic or asymptomatic frequent PVCs with or without asymptomatic VT, the sample size remains small. The control group is relatively small, because only children with consecutive Holter recordings could be included, to be able to assess the change in PVC burden. The study design did allow for multiple medication episodes per patient. The mean dosage of the AAD used in this retrospective study was relatively low; however, the mean dosage of all groups of AAD drugs were in the lower range and still flecainide shows a better result.

In addition, the self-limiting nature of this condition may have influenced the results. But there is no reason to assume that this has occurred more often in one of the groups with medication, than in the control group. The control group even had the longest interval between Holter recordings, furthermore we found no relation between age and PVC burden.

A randomized controlled trial would prevent many of these factors from interfering. Therefore, we are currently performing an open-label cross-over trial, intended to compare efficacy of flecainide to metoprolol in a population of children with more than 15% PVCs.

## Conclusions

In children with frequent symptomatic or asymptomatic PVCs with or without asymptomatic VT, the efficacy of anti-arrhythmic drug therapy is very limited. Only flecainide appears to have a statistically significant effect on reducing ventricular ectopy.

## Data Availability

All data are stored in a data safe according to the institutional regulations. Research data can be made available on request by the corresponding author.
